# Case report: Single-session double-Ilizarov lengthening technique in the treatment of a child with congenital fibular deficiency

**DOI:** 10.3389/fped.2022.952591

**Published:** 2022-07-29

**Authors:** Wen Shu, Changjie Yue, Haobo Zhong, Xin Tang

**Affiliations:** ^1^Department of Trauma Orthopedics, Liuzhou People's Hospital, Liuzhou, China; ^2^Union Hospital, Tongji Medical College, Huazhong University of Science and Technology, Wuhan, China; ^3^Department of Orthopedics, Huizhou First Hospital, Huizhou, China; ^4^Department of Orthopedics, Union Hospital, Tongji Medical College, Huazhong University of Science and Technology, Wuhan, China

**Keywords:** single session, double-Ilizarov lengthening technique, ipsilateral, congenital fibular deficiency, child

## Abstract

**Background:**

Congenital fibular deficiency is a rare disease with a broad spectrum of deformities. Associated anomalies complicate the symptoms of patients and, consequently, individualized treatments that aim at normal function and acceptable appearance.

**Case presentation:**

We present a case of congenital femoral and fibular shortening in the right lower limb with foot anomaly at school age. The patient underwent limb lengthening procedure in a single session on the right femur and tibia at the same time using a double-Ilizarov frame. The functional and cosmetic of his right lower extremity achieved a good outcome. Complications were minimal except for the superficial infection. Treatment lasted for 9.2 months, allowing for returning the patient to functional activity as soon as possible.

**Conclusion:**

A satisfactory result was obtained with limb lengthening in a single session using double Ilizarov external fixators in a school-aged patient with congenital fibular deficiency.

## Introduction

Congenital fibular deficiency (CFD) is a rare disease with an overall incidence of 7.4–20 per million live births, making it the most common long bone deficiency (1). The clinical symptoms not only present as an isolated fibular anomaly but cover a broad spectrum of deformities, including shortening of tibia and femur, ball and socket ankle, tarsal coalitions, and absent foot rays (2, 3). The limb length discrepancy, functional disability, and psychosocial and economic burden of patients and their families could make the treatment of CFD comparatively challenging when surgeons take all these into consideration (4–6).

We present a case of CFD with congenital shortening of fibula and femur with associated foot and ankle abnormalities. The patient underwent an individualized treatment characterized by an operation with single-session double-Ilizarov fixators on the ipsilateral femur and tibia. This case report aims to evaluate the outcome of this novel application, the Ilizarov technique, in the treatment of CFD. This study was approved by the ethical review board in the author's institution, and the patients' parents gave their informed consent.

## Case report

**Figure 1:**
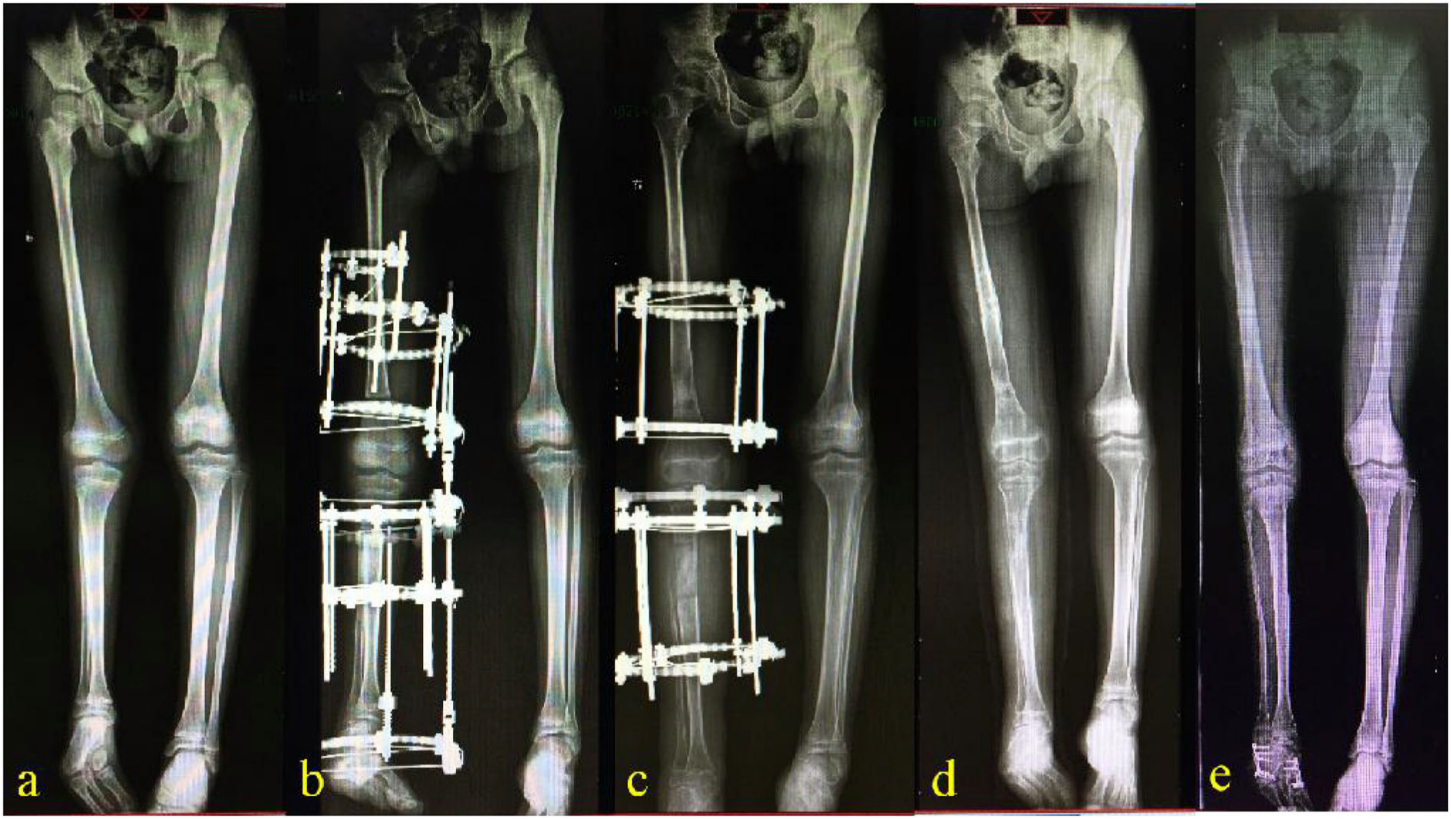
Anteroposterior X-ray view. **(a)** An 11.5-year-old boy showed congenital fibular deficiency with a total 8.8 cm shortening of the right femur and tibia, genu valgus, and was missing lateral feet in an equinovalgus position**. (b)** A double Ilizarov frame, including a femoral segment and a tibial segment, were connected with two straight/flexure device on the level of the knee joint. **(c)** The bony callus was observed almost consolidated 28 weeks after operation after lengthening, lasting for 5 weeks on the femur and 8 weeks on the tibia. **(d)** After 40 weeks of the operation, the double-Ilizarov frame was removed. **(e)** At a 3.5-year postoperative follow-up, the limb length discrepancy was 1.5 cm.

An 11.5-year-old boy was admitted to the authors' institute for shortening of the right lower extremity observed for 8 years. On physical examination, the patient had congenital shortening of the thigh and leg, genu valgus, and was missing lateral feet in an equinovalgus position. On anteroposterior (AP) radiographs, the length of the right femur and tibia were 39.0 and 30.0 cm, respectively, while on the left they were 42.4 and 35.4 cm, respectively (Figure 1a). Additionally, a three-rayed foot and talocalcaneal fusion were present. Based on history, clinical evaluation, and radiograph, the boy was diagnosed with type-1C congenital fibular deficiency (7). The short tibia in the leg gave the utmost functional defect.

He was managed by limb lengthening on the right femur and tibia in a single setting using the Ilizarov technique. Double-Ilizarov frames of appropriate size were prepared and assembled preoperatively. The femoral segment of the frame consisted of a half ring on the proximal femur, a 5/8 ring on the distal femur, and one complete ring in the middle. Lengthening of the femur was performed between the complete ring and the 5/8 ring. The tibial segment consisted of a 5/8 ring and one complete ring on the proximal tibia, a half ring for the calcaneus, and one complete ring in the middle. Lengthening of the tibia was performed between the two complete rings. The femoral and the tibial segments were connected with two straight/flexure devices on the level of the knee joint to stabilize the tension of ACL and the soft tissue of the knee joint so as to prevent against knee dislocation during lengthening (Figure 1b). Intraoperatively, blunt dissection and incisions were operated to expose ring sites when needed. The fibular osteotomy was performed transversely with a sharp osteotome. The double Ilizarov frame was applied to the right limb using percutaneous 1.8 mm K-wires and drop wires according to the Ilizarov principle. After stabilizing the external fixators and tensioning the wires, osteotomies on the right femur and tibia were performed with a bone knife after drilling. Then the wounds were closed with the application of a drainage tube.

**Figure 2:**
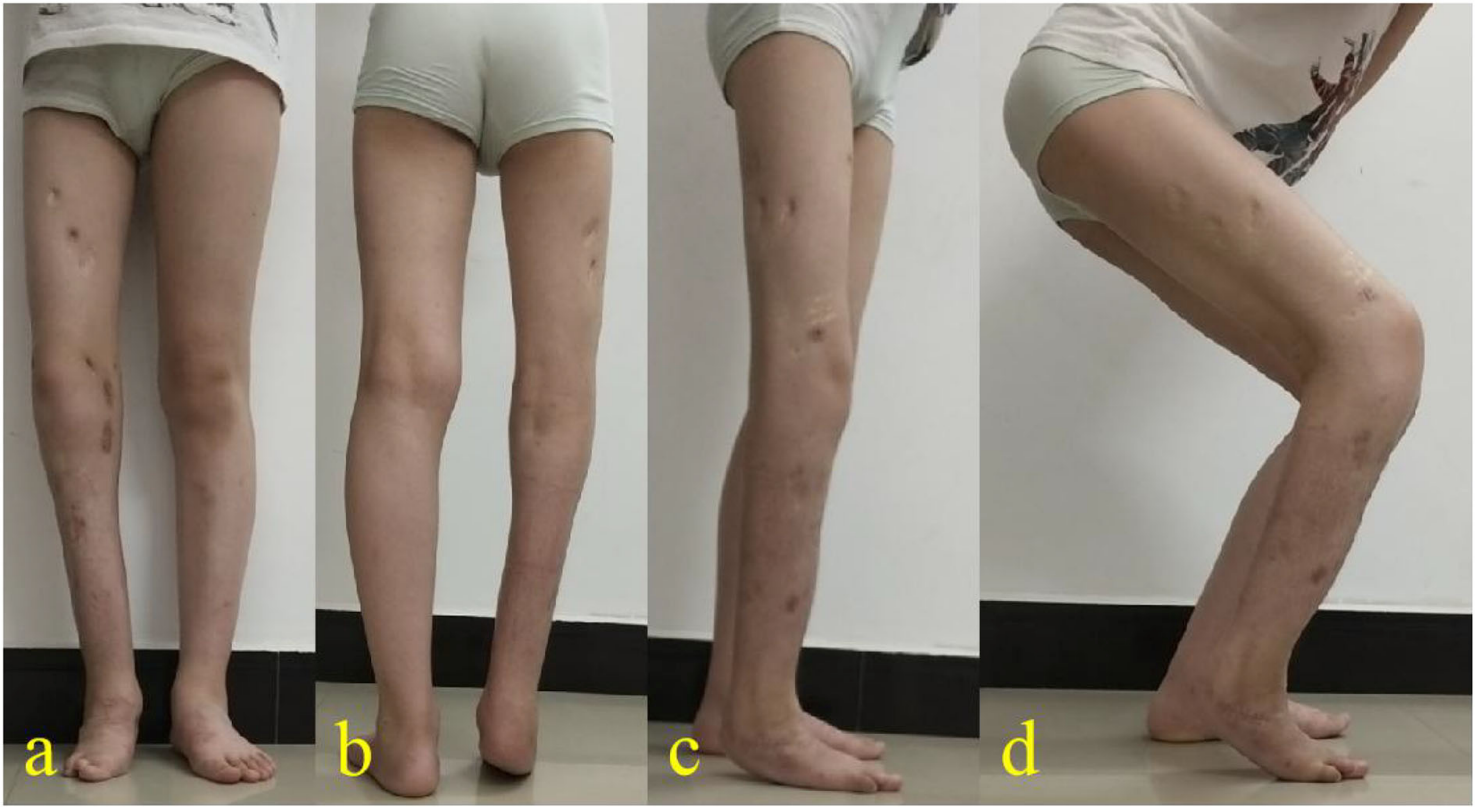
Physical examination at 3.5-year postoperative follow-up. There was a 1.5 cm limb length discrepancy cosmetic of his right lower extremity **(a,b)**. The flexion functional examination of the right knee showed moderate restriction **(c,d)**.

The duration of the surgery was 110 min, and approximately 50 ml of blood was lost during the entire surgical procedure. Oral antibiotics were prescribed, and the dressing was changed regularly. Seven days after the surgery, lengthening was started at the rate of 0.25 mm per time on the right femur and tibia four times daily. Anteroposterior and lateral radiographs were taken every 2 weeks during the lengthening process and every 4 weeks afterward. It is very important to estimate the leg length discrepancy at the end of the growth according to Paley's multiplier method, during the lengthening schedule, in stages, and during the growing period of the child (4). The lengthening lasted for 5 weeks on the femur and 8 weeks on the tibia. The half-ring of the proximal femur and half-ring of the calcaneus were removed upon complete consolidation of the bony callus at 28 weeks. The connecting rods between the femoral and tibial segment of this double-Ilizarov frame were also removed and a plantigrade foot was achieved (Figure 1c). The Ilizarov frames were completely removed at 40 weeks (Figure 1d). Superficial infection and pin-site infection were resolved after the removal of pins and a course of oral antibiotics. The knee joint range of motion was reduced to 30° on flexion compared with the normal side in physical examination, but no other delayed complication was evident. At follow-up, the limb length discrepancy was 0.3 cm, 0.96 cm, and 1.5 cm at 1 year, 2 years, and 3.5 years postoperatively, respectively (Figures 1, 2). The patient's right foot deformity corrective surgery was performed 3 years after the lengthening operation, but the equinus deformity of the foot was not part of the compensation for LDD. The LLD, at the last follow-up, was acceptable and did not require a further lengthening procedure. The flexion functional examination of the right knee showed moderate restriction (Figure 2).

## Discussion

To our knowledge, this is the first report of treatment for CFD using double Ilizarov external fixators in a single setting in a school-aged patient. There is a wide spectrum of CFD presentation, varying from mild limb inequalities to severe shortening with associated anomalies (1, 2, 8, 9). The goal of treatment in CFD is to achieve equal limb length, correct gait, and provide stable weight-bearing while walking (1). Due to its variable spectrum of presentation, individualized treatment strategies for limb lengthening or amputation should be applied (4, 6, 10). Based on both clinical deformity and anticipated treatment, new classification systems proposed by Birch et al. or Paley have been guideline methods for the surgical management of fibular deficiency in recent years (1, 7, 8). In our case, a preservable three-rayed foot was classified as type 1, and limb shortening of 8.8 cm (11.3%) was divided into type 1C, in which Birch et al. suggested that at least one lengthening was necessary (7). Intramedullary lengthening devices could be an effective option with some advantages such as acceptable appearance and low complication rate with an average of 1.2 per lengthening session. However, the application is limited to intramedullary canals and is insufficient to withstand rods (11, 12). The Ilizarov technique was introduced in China in 1990, and some researchers preferred it because it maintains the mechanical axis with simultaneous correction of limb length discrepancy and ankle or foot deformities (13–16).

There are several published series of lengthening following the Ilizarov technique in CFD, including two or more lengthening sessions during growth (6, 11, 13–15). Moreover, the final lengthening may be performed at skeletal maturity to correct prediction errors of limb length discrepancy (1). Concerns regarding the high complication rate, the prolonged hospital stay, and psychosocial impact on the patient and family in repeated lengthening have been highlighted by limb reconstruction surgeons (5, 16, 17). A similar method has been reported by Bishay, who achieved good results in all eight patients with type-1 fibular deficiency (18). The application was limited to patients older than 14 years with stable hip, knee, and ankle joints but with no coxa vara, genu valgus, or foot anomaly. The age of the current case was 11.5 years old. However, this method should not be recommended for patients at 5–10 years old because of the increased risk of complications and relapse. Also, Bishay reported a series with femoral shortening of 2–5cm and recommended femoral lengthening simultaneously (18).

This study demonstrated that satisfactory results could be obtained with limb lengthening in a single setting using double Ilizarov external fixators in school-age children. The lengthening index (total treatment time in months per cm of lengthening) was 1.01, while it was reported to be averaged 1.10 to 1.50 in multiple sessions (6, 13–16). But it should be noticed that this superiority may be partly attributed to the shorter lengthening target (9.1 cm) and the less severe type of deformity. There is always an increased risk for possible nerve traction in a simultaneous lengthening of the femur and tibia, but a possible decrease in the rate of lengthening can be an option. Although we did not observe this in our case, it might be because of a relatively minimal possible lengthening target and a gradual distraction process. Also, fewer complications might be attributed to careful nursing care and parents' education. This treatment had certain drawbacks, such as stiffness and loss of knee range of motion, but those were minor and acceptable considering the achievement of acceptable length and acceptable normal weight-bearing. In practice, the involvement of the child in the decision-making process and fully informed consent might contribute to cooperation in such a long treatment period.

Despite the initial success, a single case with a 3.5-year follow-up provides insufficient data to establish the potential superiority of our strategy over others. Future studies comparing the efficacy of double lengthening of a single setting and isolated lengthening of multiple settings with the Ilizarov method could provide better evidence. However, a satisfactory result was obtained with limb lengthening in a single session using double Ilizarov external fixators in a school-aged patient with CFD.

## Data Availability Statement

The original contributions presented in the study are included in the article/supplementary material, further inquiries can be directed to the corresponding author/s.

## Ethics Statement

The studies involving human participants were reviewed and approved by the Ethics Committee of Tongji Medical College, Huazhong University of Science and Technology (IORG No: IORG0003571). Written informed consent to participate in this study was provided by the participants' legal guardian/next of kin. Written informed consent was obtained from the individual(s), and minor(s)' legal guardian/next of kin, for the publication of any potentially identifiable images or data included in this article.

## Author contributions

CY was involved in data collection and follow-up assessments. XT and HZ were responsible for literature search, study design, and finalized the manuscript. WS and CY drafted the manuscript. All authors contributed to the article and approved the submitted version.

## Conflict of interest

The authors declare that the research was conducted in the absence of any commercial or financial relationships that could be construed as a potential conflict of interest.

## Publisher's note

All claims expressed in this article are solely those of the authors and do not necessarily represent those of their affiliated organizations, or those of the publisher, the editors and the reviewers. Any product that may be evaluated in this article, or claim that may be made by its manufacturer, is not guaranteed or endorsed by the publisher.
